# The importance of illness severity and multimorbidity in the association between mental health and body weight in psoriasis: Cross‐sectional and longitudinal analysis

**DOI:** 10.1002/ski2.117

**Published:** 2022-04-19

**Authors:** Neli T. Pavlova, Rona Moss‐Morris, Catherine Smith, Ewan Carr, Lauren Rayner, Federica Picariello

**Affiliations:** ^1^ Health Psychology Section Psychology Department Institute of Psychiatry Psychology and Neuroscience King's College London London UK; ^2^ Guy's and St Thomas' NHS Foundation Trust St John's Institute of Dermatology London UK; ^3^ Department of Biostatistics and Health Informatics Institute of Psychiatry Psychology and Neuroscience King's College London London UK; ^4^ Department of Psychological Medicine Institute of Psychiatry Psychology and Neuroscience King's College London London UK

## Abstract

**Background:**

High body weight is common in psoriasis and is associated with depression and anxiety. Past studies are mostly cross‐sectional and may underestimate the role of demographic and illness‐related factors in the association between mental health and body weight in psoriasis.

**Objectives:**

This study explored the association between depression and anxiety with waist circumference and body mass index (BMI) cross‐sectionally and at 12 months follow‐up, adjusting for demographic and illness‐related factors in people with psoriasis.

**Method:**

Routine psoriasis care data were combined with data on depression and anxiety from a large specialist psoriasis centre. The analytical samples consisted of patients with complete data on either waist circumference (*N* = 326 at time 1; *N* = 191 at follow‐up) or BMI (*N* = 399 at time 1; *N* = 233 at follow‐up) and corresponding mental health, demographic, and illness‐related information. Associations between weight‐related outcomes and mental health variables were assessed at time one and at 12 months follow‐up, after adjusting for demographic and illness‐related factors.

**Results:**

We found no evidence of associations between mental health and waist circumference or BMI, after adjusting for age, gender and illness‐related factors. Higher age, male gender and illness‐related factors, specifically multimorbidity and psoriasis severity, were positively associated with waist circumference and BMI at both time points.

**Conclusion:**

This study revealed the important role of factors related to illness severity in body weight in psoriasis. The contribution of depression and anxiety to weight was not observed here likely due to the sample and methodology used. Future work should explore other psychosocial factors such as weight‐related attitudes and emotional eating in the context of weight in psoriasis, to help inform the development of successful weight‐management treatments.

1

**What is already known about this topic?**

- High body weight, common in psoriasis, is associated with depression and anxiety. Depression and anxiety affect disproportionally women with psoriasis compared to men. Multimorbidity and greater psoriasis severity may increase the psychological burden on patients' lives. Despite that, current research is mostly cross‐sectional and underestimates the role of demographic and illness‐related factors in the association between depression and anxiety with body weight in psoriasis.

**What does this study add?**

- We found no evidence of associations between depression and anxiety with body weight after adjusting for demographic and illness‐related factors. Further research needs to investigate the relationship between depression and anxiety with body weight in a more robust study design and explore the underlying cognitive and behavioural components that may help to explain the high incidence of obesity in psoriasis.

## 2 INTRODUCTION

Psoriasis is a chronic debilitating inflammatory systemic condition that affects 2%–9% of the world's population.^1^ One‐third of people living with psoriasis have comorbid obesity,^2^ defined as a body mass index (BMI) of ≥30 kg/m.^3^ Psoriasis and obesity frequently co‐occur with depression and anxiety in a bi‐directional fashion, where obesity may drive mental health issues and vice versa.^4^, ^5^, ^6^, ^7^, ^8^ This co‐occurrence may be due to the physical and emotional toll that psoriasis and comorbid obesity have on patients' lives.^9^, ^10^ Pain, discomfort, difficulty performing daily tasks and low self‐esteem are common symptoms of both psoriasis and obesity.^10^, ^11^

Obesity is frequently considered in the context of high BMI, an index for relating weight to height, as means of determining whether a person's weight is healthy.^12^ To date, it is the main indicator of healthy weight considered by studies that investigated the relationship between obesity and mental health in psoriasis.^7^ However, BMI does not differentiate between body fat mass and muscle mass.^13^ Hence, it is not a reliable indicator of actual body fat mass.^14^ This is crucial because a higher percentage of visceral fat is associated with poor physical and mental health outcomes.^15^ Incorporating a more accurate indicator of body fat such as waist circumference, alongside BMI, may allow for more precise monitoring of the association between obesity and comorbid medical and mental health conditions, including anxiety and depression.^6^, ^7^

Common psoriasis‐ and obesity‐associated comorbidities such as cardiometabolic, kidney and gastrointestinal diseases are also overlooked in the association between mental health and obesity in psoriasis.^16^, ^17^ These co‐morbid conditions may increase the psychological burden on patients' lives.^10^ They ought to be considered when determining the association between mental health and obesity in psoriasis. Important nuances related to demographic factors should also be factored in. Depression and anxiety affect disproportionately women with psoriasis compared to men.^18^ Higher BMI has been linked to depression in females (24.5%, 95% CI = 17.2, 33.5), but not in males (11.6%, CI = 6.5, 19.7).^19^, ^20^, ^21^ Age is another important demographic factor that should be considered. As age increases, the association between waist circumference and BMI with depression becomes stronger in the general population.^22^ It is yet to be determined whether there is a link between body weight and mental health in psoriasis and if it is gender‐ or age‐dependent.

To recognize the complex relationship between obesity and mental health in psoriasis, we need to look beyond general weight loss models that focus on dietary habits and physical activity.^7^ Tailored illness‐specific interventions that recognize the role of demographic, illness‐related and mental health factors have been linked to better weight outcomes in other patient populations.^19^, ^23^ Depression and anxiety can be significant barriers to engagement with and adherence to weight‐loss recommendations, and they can also prevent long‐term maintenance of weight loss.^24^, ^25^ Psoriasis management guidelines recommend a holistic approach in which patients' treatment goals include both weight management and improved mental health.^14^ As a result, it is critical to build a better understanding of the role of depression and anxiety in psoriasis' body weight, taking into consideration nuances related to demographic factors and illness severity. This would allow for a more tailored and effective approach to weight reduction that addresses the multifaceted needs of people living with psoriasis, ultimately resulting in better patient outcomes.^7^

This study used data collected as part of routine specialist care to gain a more robust understanding of the role of depression and anxiety in psoriasis patients' weight‐related outcomes (including waist circumference and BMI), considering the limitations of earlier studies. First, the relationship between illness‐related factors and body weight outcomes in psoriasis were assessed to determine if they may be potential confounds in the relationship between depression or anxiety and weight‐related outcomes. Secondly, we assessed the relationship between mental health and weight outcomes when adjusting for both demographic and illness related factors cross‐sectionally. We also explored the longitudinal relationship between mental health at time one with body weight outcomes measured at 12 months follow‐up. The following hypotheses were formulated.

1. Multimorbidity and more severe psoriasis at time one will be positively associated with waist circumference and BMI (i) at time one and (ii) at 12 months follow‐up; after adjusting for demographic variables (age, gender and ethnicity).
2. Depression and anxiety at time one will be positively associated with waist circumference and BMI (i) at time one and (ii) at 12 months follow‐up; after adjusting for demographic and illness‐related variables (psoriasis severity, comorbidities, and psoriasis treatment).
3. Gender will moderate the relationship between depression and anxiety at time one with waist circumference and BMI (i) at time one and (ii) at 12 months follow‐up; where the relationship between depression and anxiety with waist circumference and BMI at both time one and 12 months follow‐up will be greater for women than men.

## 3 METHODS

### 3.1 Patients

Patients were recruited between 2014 and 2020 during routine dermatology outpatient visits at a large specialist psoriasis centre serving London and South East England. Routine psoriasis treatment data from 727 patients were combined with depression and anxiety screening data from the same patients collected through the Integrating Mental and Physical Healthcare: Research Training and Services (IMPARTS) screening programme. The IMPARTS programme provides a multifaceted platform of clinical and research services to assist in the integration of mental healthcare into routine care for patients with physical health conditions and is further described elsewhere.^26^ At each appointment, participants filled out self‐report measures of depression and anxiety using a tablet. As part of their usual care, they were then clinically assessed by their doctor. Patients could have multiple appointments during the follow‐up period (between 1 and 23). To maximize the use of available data, three analytical samples were defined (Table 1) all of which had complete data on either BMI or waist circumference, on covariates (e.g., demographic, illness‐related), and mental health variables (depression and anxiety). Patients with incomplete data on BMI or waist circumference, covariates (e.g., demographic, illness‐related), or mental health variables (depression and anxiety) were excluded from the analytical samples.

**TABLE 1:** The analytical samples in the analysis

Description	Identifier	
	At time one	At follow‐up
Patients with complete waist circumference data	A	B
Patients with complete BMI data	C	D
Patients with missing waist circumference and/or BMI data	E	F

Since IMPARTS data collection is part of routine clinical care, formal consent to participate is not required. Patients are told that their anonymized data may be used for research purposes (REC reference: 12/SC/0422) and that they can opt‐out at any time. This study followed the Strengthening the Reporting of Observational Studies in Epidemiology (STROBE) reporting guideline^27^ (See supplementary material C).

## 4 MEASURES

### 4.1 Weight‐related outcomes

Weight‐related data (waist circumference [cm], height [m] and weight [kg]) are routinely collected during dermatology appointments. BMI was calculated as weight in kg divided by height in m^2^. BMI categories were defined as healthy weight (BMI between 18.5 and 24.9 kg/m^2^), overweight (BMI between 25.0 and 29.9 kg/m^2^), obese (BMI between 30.0 and 40.0 kg/m^2^), and morbidly obese (BMI ≥ 40.0 kg/m^2^).^12^ The threshold for healthy waist circumference was defined as < 94 cm and 80 cm for men and women, respectively.^28^ Abdominal obesity was considered as ‘present’ if the threshold was reached or exceeded.

The term ‘time one’ refers to patients' first appointment in the database. Most participants will have had appointments before this time, preceding the data collection period. Based on data availability and completeness, a 12‐month follow‐up appointment was chosen to extract follow‐up data (the appointment closest to 12 months was selected, within a window of 10 to14 months).

### 4.2 Mental health

The two‐item and nine‐item Patient Health Questionnaire (PHQ‐2, PHQ‐9) were used to screen for probable major depressive disorder (herein, depression).^29^, ^30^ The two‐item and seven‐item Generalised Anxiety Disorder Scale‐7 (GAD‐2, GAD‐7) were used to screen for probable generalized anxiety disorder (herein, anxiety).^31^ Details can be found in the eMethods (see supplementary material B).

### 4.3 Covariates

Covariates were selected based on theory and prior evidence and measured at time one. Demographic covariates included age, gender and ethnicity. Because of the scarcity of routine care data from patients belonging to minority ethnic groups (19.9% of the overall patient sample), ethnicity was dichotomized into (i) White and (ii) Black, Asian, and minority ethnic (BAME). Illness‐related factors included the total number of physical comorbidities,^10^ current treatment type^32^ and psoriasis severity.^33^ The number of comorbidities was calculated using routine data on the presence or absence of seven psoriasis‐ and obesity‐related physical comorbidities (hypertension, type 2 diabetes, liver disease, bone disease, asthma, psoriatic arthritis and cancer).^34^, ^35^ A total count of present comorbid conditions was also computed (range between 0 to 7). While comorbid depression and anxiety were recorded, these were removed from the count of comorbidities to avoid overlap with the variables of interest, PHQ and GAD. Treatment was dichotomized as either (i) systemic therapy (treatment for psoriasis taken orally, subcutaneous, or intravenously) or (ii) no systemic treatment. This approach was taken in line with consensus that systemic therapies generally indicate more severe disease^36^ requiring more intensive treatment compared to topicals.^37^ Psoriasis severity was measured with the Psoriasis Area Severity Index (PASI), a clinician‐rated scale for measuring the severity of psoriatic lesions based on area coverage and plaque appearance.^38^

### 4.4 Statistical analyses

There were four stages to the analysis.

First, to investigate the impact of missing data, patients with and without outcome data (waist circumference and BMI) at time one and follow‐up were compared for demographic, illness‐related and mental health variables (independent‐measures *t*‐test for continuous variables and χ^2^ test for categorical variables; see Tables S1 and S2). Patients with available weight‐related data at time one versus those with available data at follow‐up were also compared (Table 3).

Second, we used correlations, χ^2^ tests and *t*‐tests to explore bivariate associations between demographic, illness‐related, and mental health variables, and outcomes. This was done to assess associations between (i) variables measured at time one; and (ii) variables measured at time one and outcomes measured at follow‐up (see Table S3).

Third, predictors of waist circumference and BMI were evaluated using multiple linear regression models. We estimated models to test whether depression and anxiety at time one were associated with weight‐related outcomes (i) at time one and (ii) at 12 months follow‐up. All models were adjusted for demographic and illness‐related covariates measured at time one. In the models using weight‐related outcomes at 12 months follow‐up, we did not adjust for waist circumference and BMI at time one as the preliminary analyses showed no clinically meaningful weight changes (i.e., at least a 5% reduction in weight from baseline level).^39^ Between time one and 12 months follow‐up, waist circumference showed a mean reduction of 1.8% (−0.82 cm, *p* = 0.163) and BMI a mean reduction of 2.3% (−0.21 kg/m^2^, *p* = 0.088).

Fourth, using interaction terms and stratified regression models, we tested whether gender moderated the associations between depression and anxiety measured at time one with weight‐related outcomes measured at (i) time one and (ii) 12 months follow‐up. We included interaction terms (e.g., female gender × anxiety) and main effects into the above regression models. The statistical significance of the interaction was tested with likelihood‐ratio tests.

Model fit was evaluated using a ΔF‐statistic. Improvement in explained variance with each regression step was calculated using adjusted Δ*R* ^2^. Statistical significance level was assumed at *p* ≤ 0.05. All analyses were conducted in Stata 16.^40^

## 5 RESULTS

### 5.1 Sample characteristics

There were 326 and 191 patients with available weight circumference data at time one and follow‐up, respectively; and 399 and 233 patients with available BMI data at time one and follow‐up, respectively. Of these, just over half (54%) had abdominal obesity based on waist circumference at time one. Most patients (75%) at time one were overweight, with 19% and 18% living with obesity or morbid obesity based on BMI, respectively. At time one, depressed and anxious patients reported higher PASI and fewer comorbidities (likely due to the little variability in the number of comorbidities) compared to those not depressed and not anxious (see Table S8).

### 5.2 Missing data

We compared samples with available waist circumference and BMI data to those with missing waist circumference and BMI data on a variety of demographic, illness severity, and mental health measures at time one and at 12 months follow‐up. This consideration allowed us to ascertain if missing data influenced the conclusions on the associations between mental health and body weight (i) at time one and (ii) at 12 months follow‐up.

When looking at availability of weight‐related outcome data at time one, males were significantly more likely to have available waist circumference data compared to females (males = 60%; females = 40%, *p* = 0.008). At time one, patients who were not depressed were significantly more likely to have available BMI data than patients who presented as depressed (68% vs. 32%, *p* = 0.004). There were no other differences between those with and without BMI and waist circumference data at time one (see Tables S1 and S2).

When looking at availability of weight‐related outcome data at 12 months follow‐up, patients with missing waist circumference and BMI data at follow‐up, were significantly less likely to be on systemic treatment for psoriasis compared to those not missing weight‐related outcome data (27% vs. 73%, *p* = 0.030; see Tables S1 and S2).

Furthermore, patients with available waist circumference and BMI data at time one had significantly higher PASI scores than patients with available waist circumference and BMI at 12 months follow‐up. Table 2 summarises the patterns of missing and analytical data. Table 3 shows the demographic, illness‐related and mental health characteristics of the sample at time one and at 12 months follow‐up.

**TABLE 2:** Participant data (*n*) and exclusions for each analytic sample

Excluded – waist circumference/BMI	Excluded comorbidities	Excluded treatment type	Excluded PASI
Time one sample A (*n* = 326)			
*n* = 328, 45.1%	*n* = 54, 13.2%	*n* = 14, 3.4%	*n* = 5, 1.2%
Follow‐up sample B (*n* = 191)			
*n* = 502, 69.0%	*n* = 25, 11.2%	*n* = 7, 3.1%	*n* = 2, 0.9%
Time one sample C (*n* = 399)			
*n* = 247, 34.0%	*n* = 57, 11.9%	*n* = 16, 3.3%	*n* = 8, 1.7%
Follow‐up sample D (*n* = 233)			
*n* = 450, 62.0%	*n* = 34, 12.4%	*n* = 8, 2.9%	*n* = 2, 0.7%

*Note*: Total *N* on IMPARTS database = 727.

**TABLE 3:** Demographic, illness‐related and mental health characteristics of the analytical samples at time one and at 12 months follow‐up

		Time one (the first available data entry)		Follow‐up (12 months later)		Time one versus follow‐up	
Variable		Sample A with information on waist circumference (*N* = 326)	Sample C with information on BMI (*N* = 399)	Sample B with information on waist circumference (*N* = 191)	Sample D with information on BMI (*N* = 233)	Sample A versus Sample B	Sample C versus Sample D
Gender, *n* (%)	Female	128 (31.2%)	168 (35.2%)	69 (30.9%)	89 (32.5%)	*χ* ^2^ = 0.001, *p* = 0.943	*χ* ^2^ = 0.55, *p* = 0.458
	Male	282 (68.8%)	310 (64.8%)	154 (69.1%)	185 (67.5%)		
Age (year), mean (SD)		46.04 (13.6)	45.7 (13.3)	45.6 (12.7)	45.3 (12.9)	*t*(631) = −0.44, *p* = 0.662	*t*(750) = −0.33, *p* = 0.742
Ethnicity *n* (%)	White	333 (81.2%)	385 (80.5%)	178 (79.8%)	216 (78.8%)	*χ* ^2^ = 0.18, *p* = 0.670	*χ* ^2^ = 0.32, *p* = 0.573
	Black and ethnic minorities	77 (18.8%)	93 (19.5%)	45 (20.2%)	58 (21.2%)		
Treatment n (%)	Systemic treatment, yes	232 (60.1%)	270 (58.7%)	144 (66.7%)	158 (59.4%)	*χ* ^2^ = 2.54, *p* = 0.111	*χ* ^2^ = 0.03, *p* = 0.853
	No systemic treatment, yes	154 (39.9%)	190 (41.3%)	72 (33.3%)	108 (40.6%)		
PASI, mean (SD)		4.7 (5.1)	4.8 (4.8)	3.4 (4.4)	3.7 (4.0)	***t*(614) = **−3.02, *p* = 0.0027*	***t*(738) = **−3.16, *p* = 0.0016*
Comorbidities *n* (%)	Diabetes	26 (6.3%)	32 (6.7%)	13 (5.8%)	19 (6.9%)	*χ* ^2^ = 0.07, *p* = 0.798	*χ* ^2^ = 0.02, *p* = 0.900
	Hypertension	82 (20.0%)	110 (23.0%)	67 (24.5%)	44 (19.7%)	*χ* ^2^ = 0.01, *p* = 0.935	*χ* ^2^ = 0.20, *p* = 0.654
	Presence of liver disease, *n* (%)	42 (10.2%)	45 (9.4%)	24 (10.8%)	31 (11.3%)	*χ* ^2^ = 0.04, *p* = 0.838	*χ* ^2^ = 0.692, *p* = 0.406
Total number of comorbid medical conditions, mean (SD)		1.02 (1.29)	1.02 (1.32)	0.93 (1.34)	1.10 (1.35)	*t*(552) = −0.75, *p* = 0.451	*t*(659) = 0.74, *p* = 0.461
Depression (PHQ), *n* (%)	Depressed	86 (20.9%)	81 (16.9%)	34 (15.2%)	42 (15.3%)	*χ* ^2^ = 3.09, *p* = 0.079	*χ* ^2^ = 0.33, *p* = 0.564
	Not depressed	324 (79.1%)	397 (83.1%)	189 (84.8%)	232 (84.7%)		
Anxiety (GAD), *n* (%)	Anxious	71 (17.3%)	72 (15.1%)	30 (13.5%)	32 (11.7%)	*χ* ^2^ = 1.61, *p* = 0.205	*χ* ^2^ = 1.67, *p* = 0.196
	Not anxious	339 (82.7%)	406 (84.9%)	193 (86.5%)	242 (88.3%)		
Abdominal obesity, n (%)	Obese	225 (54.8%)	347 (72.6%)	136 (61.0%)	208 (75.9%)		
	Non‐obese	185 (45.2%)	131 (27.4%)	87 (39.0%)	66 (24.1%)	*χ* ^2^ = 2.20, *p* = 0.138	*χ* ^2^ = 0.99, *p* = 0.319

**p ≤ 0.001; *p < 0.05.

### 5.3 Bivariate associations between demographic, illness‐related and mental health (explanatory variables), and waist circumference and BMI (outcome variables)

Age and the total number of comorbidities were significantly positively correlated with waist circumference and BMI at time one. Significantly larger waist circumference was observed among males compared to females, but no difference was observed in BMI by gender. PASI scores at time one were significantly positively correlated with higher BMI, but not waist circumference, at 12 months follow‐up (see Table S3).

### 5.4 Associations between demographic, illness‐related and mental health (explanatory variables), and waist circumference and BMI (outcome variables) at time one

Table 4 presents associations of mental health at time one with waist circumference and BMI at time one, adjusting for demographic and illness‐related variables at time one. In the final models, there was no evidence for a significant association between depression and anxiety with waist circumference or BMI. Age (*B* = 0.20, 95% CI 0.05, 0.34; *B* = 0.06, 95% CI 0.02, 0.11, respectively) and the total number of comorbid medical conditions (*B* = 2.06, 95% CI 0.63, 3.48; *B* = 0.87, 95% CI 0.42, 1.32, respectively) remained significantly associated with both waist circumference and BMI. Male gender was associated with larger waist circumference (*B* = 6.48, 95% CI 2.77, 10.18), but not BMI.

**TABLE 4:** Regression models of waist circumference and BMI as outcomes at time one

	Waist circumference *at time one* (*N* = 326)		BMI *at time one* (*N* = 399)	
	*B*	95% CI	*B*	95% CI
	Step 1, *R* ^2^ = 7.16%		Step 1, *R* ^2^ = 4.32%	
Age	0.23**	**0.12**–**0.35**	0.09**	**0.05**–**0.12**
Gender, male	6.27**	**2.94**–**9.60**	0.16	−0.89–1.21
Ethnicity, White	0.74	−3.24–4.71	0.65	−0.60–1.94
	Step 2, *R* ^2^ = 11.97%		Step 2, *R* ^2^ = 9.92%	
Age	0.20*	**0.06**–**0.35**	0.06*	**0.02**–**0.11**
Gender, male	6.33**	**2.64**–**10.02**	−0.22	−1.38–0.95
Ethnicity, White	1.55	−2.66–5.76	0.88	−0.46–2.23
Comorbidities, total	1.93*	**0.51**–**3.34**	0.85**	**0.40**–**1.30**
Systemic treatment, present	1.49	−1.98–4.95	0.81	−0.30–1.93
PASI	0.32	−0.02–0.65	0.11	−0.004–0.23
	Step 3, *R* ^2^ = 12.57%		Step 3, *R* ^2^ = 10.17%	
Age	0.20*	**0.05**–**0.34**	0.06*	**0.02**–**0.11**
Gender, male	6.50**	**2.80**–**10.19**	−0.16	−1.33‐1.02
Ethnicity, White	1.75	−2.46–5.97	0.92	−0.42–2.27
Comorbidities, total	2.06*	**0.64**–**3.48**	0.88**	**0.42**–**1.33**
Systemic treatment, present	1.79	−1.69–5.28	0.89	−0.24–2.01
PASI	0.26	−0.08–0.61	0.10	−0.02–0.22
Depression, present	3.40	−1.14–7.95	0.84	−0.75–2.44
	Step 4, *R* ^2^ = 12.58%		Step 4, *R* ^2^ = 10.37%	
Age	0.20*	**0.05**–**0.34**	0.06*	**0.02**–**0.11**
Gender, male	6.48**	**2.77**–**10.18**	−0.19	−1.36–0.99
Ethnicity, White	1.75	−2.47–5.97	0.86	−0.49–2.22
Comorbidities, total	2.06*	**0.63**–**3.48**	0.87*	**0.42**–**1.32**
Systemic treatment, present	1.78	−1.71–5.27	0.87	−0.26–1.99
PASI	0.26	−0.08–0.60	0.10	−0.02–0.21
Depression, present	3.90	−2.87–10.66	1.66	−0.66–3.98
Anxiety, present	−0.69	−7.69–6.31	−1.15	−3.54–1.23

**p ≤ 0.001; *p < 0.05.

The models for time one waist circumference (*F* (9, 316) = 5.28, *p* ≤ 0.001) and BMI (*F* (9, 389) = 5.01, *p* **≤ ** 0.001) were statistically significant and explained a total of 13% and 10% of variance in waist circumference and BMI, respectively. Demographic and illness‐related variables explained the larger proportion of the variance in both weight‐related outcomes.

### 5.5 Associations between time one explanatory variables and waist circumference and BMI measured at 12 months follow‐up

Table 5 presents associations of mental health at time one with waist circumference and BMI at 12 months follow‐up, adjusting for demographic and illness‐related variables at time one. We found no evidence of associations between mental health at time one and weight‐related outcomes at 12 months follow‐up. The total number of comorbid medical conditions was associated with both waist circumference (*B* *=* 2.28, 95% CI 0.60, 3.95) and BMI (*B* *= *0.66, 95% CI 0.02, 1.30) at follow‐up. Age (*B* = 0.28, 95% CI 0.10, 0.45), male gender (*B* = 6.85, 95% CI 2.50, 11.20), and PASI (*B* *=* 0.94, 95% CI 0.49, 1.38), remained significantly associated with waist circumference at 12 months follow‐up (Table 5).

**TABLE 5:** Regression models of waist circumference and BMI as outcomes at 12 months follow‐up

	Waist circumference *at follow‐up* (*N* = 191)		BMI *at follow‐up* (*N* = 233)	
	*B*	95% CI	*B*	95% CI
	Step 1, *R* ^2^ = 17.44%		Step 1, *R* ^2^ = 4.87%	
Age	0.39**	**0.25**–**0.54**	0.09*	**0.03**–**0.14**
Gender, male	7.39**	**3.44**–**11.34**	0.48	−0.98–1.93
Ethnicity, White	2.47	−2.14–7.08	1.07	−0.59–2.73
	Step 2, *R* ^2^ = 26.36%		Step 2, *R* ^2^ = 7.50%	
Age	0.28*	**0.10**–**0.45**	0.06	−0.01–0.12
Gender, male	6.67*	**2.34**–**11.01**	0.31	−1.34–1.97
Ethnicity, White	4.21	−0.64–9.07	1.23	−0.57–3.02
Comorbidities, total	2.21*	**0.57**–**3.85**	0.62*	**−0.01**–**1.26**
Systemic treatment, present	0.26	−4.01–4.53	0.54	−1.01–2.10
PASI	0.96**	**0.52**–**1.41**	**0.07**	**−0.12**–**0.25**
	Step 3, *R* ^2^ = 26.97%		Step 3, *R* ^2^ = 7.59%	
Age	0.28*	**0.10**–**0.45**	0.06	−0.01–0.12
Gender, male	6.86*	2.52–**11.20**	0.33	−1.33–2.00
Ethnicity, White	4.75	**−0.18**–9.67	1.27	−0.54–3.08
Comorbidities, total	2.29*	0.65–**3.93**	0.63*	**−0.003**–**1.27**
Systemic treatment, present	0.22	**−4.05**–4.48	0.54	−1.02–2.09
PASI	0.94**	0.49–**1.38**	0.06	−0.13–0.25
Depression, present	3.55	−2.11–9.21	0.54	−1.64–2.72
	Step 4, *R* ^2^ = 29.97%		Step 4, *R* ^2^ = 7.98%	
Age	0.28*	**0.10**–**0.45**	0.06	−0.004–0.12
Gender, male	6.85*	**2.50**–**11.20**	0.35	−1.31–2.02
Ethnicity, White	4.74	−0.21–9.68	1.26	−0.55‐3.06
Comorbidities, total	2.28*	**0.60**–**3.95**	0.66*	**0.02**–**1.30**
Systemic treatment, present	0.21	−4.07–4.49	0.59	−0.97–2.16
PASI	0.94**	**0.49**–**1.38**	0.06	−0.13–0.25
Depression, present	3.82	−4.77–12.41	−0.47	−3.46–2.53
Anxiety, present	−0.37	−9.15–8.41	1.64	−1.70–4.98

**p ≤ 0.001; *p < 0.05.

The overall models for waist circumference (*F* (9, 181) = 7.87, *p* **≤** 0.001) and BMI (*F* (9, 223) = 2.33, *p* **= **0.02) at 12 months follow‐up were significant, explaining a total of 30% and 8% of variance in waist circumference and BMI, respectively. Demographic and illness‐related variables explained the larger proportion of the variance in both weight‐related outcomes.

### 5.6 Gender as moderator

There was no evidence of an interaction effect between gender and mental health on waist circumference and BMI at time one and follow‐up in any of the models (see Tables S4–S7).

## 6 DISCUSSION

This study drew on routine care data to gain a better understanding of the role of depression and anxiety in people with psoriasis' weight‐related outcomes. It adds to the small number of studies in this area by examining the association between mental health and body weight using waist circumference as a valid marker of abdominal and visceral fat,^41^ in addition to more commonly used BMI. The study improves on prior cross‐sectional research by adjusting the analysis for several important demographic and illness‐related factors, which could confound links between mental health and weight‐related outcomes. In addition, this is the first study to examine these relationships over time.

Contrary to hypotheses and previous studies,^4^, ^5^, ^7^ we found no evidence of associations between depression and anxiety at time one with weight‐related outcomes cross‐sectionally or 12 months later, when adjusting for a wide range of demographic and illness‐related factors. In line with hypotheses, we found positive associations between the number of comorbidities and weight‐related outcomes cross‐sectionally and 12 months later. Greater psoriasis severity at time one, measured by PASI, was also positively associated with waist circumference 12 months later. In terms of demographics, older age was positively associated with waist circumference cross‐sectionally and 12 months later, and with BMI cross‐sectionally only. Male gender was associated with waist circumference cross‐sectionally and 12 months later, but not with BMI. Contrary to expectations, there was no moderation effect of gender on the relationship between mental health and weight outcomes.

### 6.1 In comparison to previous literature

Our cross‐sectional findings were inconsistent with previous cross‐sectional studies that reported positive associations between depression and anxiety with weight‐related outcomes in psoriasis.^7^ These inconsistencies may be explained by previous studies not adjusting for the role of psoriasis severity and medical comorbidities.^4^, ^7^, ^42^ They may also relate to the fact that in this study we also used a cut‐off to define probable caseness of depression and anxiety.^30^, ^31^ Continuous measures were adopted in other research that indicated a link between depression and weight outcomes.^43^ It may be that distress, rather than clinically defined categories of anxiety and depression, is associated with weight outcomes.^67^, ^68^

It is also possible that both non‐response and attrition biases may have played a role in the lack of association observed between depression and anxiety with weight‐related outcomes.^64^, ^65^ Nearly half of participants at time one were missing information on weight‐related outcomes at follow‐up. Our findings indicated that people without depression were significantly more likely to have data on BMI at time one in comparison to people with depression. There was also a large amount of missing data at follow‐up, which may have obscured the association between depression and anxiety with weight‐related outcomes.

The evidence of a positive association between older age and waist circumference at both time points, and with BMI cross‐sectionally only, agrees with previous studies.^44^, ^45^ Elderly people living with psoriasis and obesity are certainly at risk of added disability and morbidity^44^ which should be of a particular focus in future studies. We found that male gender was associated with waist circumference cross‐sectionally and at 12 months follow‐up which is consistent with previous observations that men have a relatively more central fat distribution than females.^47^ This finding confirms that using solely BMI to monitor impact of increasing weight status may lead to underestimation of the associated health burden.^46^

The findings that both psoriasis severity and co‐morbidities are related to waist circumference and BMI are consistent with previous studies.^2^, ^48^ These relationships are likely to be complex and bidirectional. Multimorbidity and greater severity of psoriasis can exacerbate the physical and psychological burden of psoriasis and act as a barrier to heathy behaviours,^10^ as well as contribute to depression.^49^ Patients dealing with multimorbidity may be more prone to unhelpful coping behaviours such as social withdrawal and emotional eating, both of which have been linked to obesity and depression.^50^, ^51^, ^52^ It is also important to consider potential mobility impairments due to multimorbidity. Disability may act as an additional barrier to healthy behaviours,^53^ leading to reduced activity levels.^54^

Equally, excess weight may contribute to multimorbidity and more severe disease as it interferes with treatment response.^2^ People with a high BMI and multiple medical conditions are more likely to report worsened psoriasis symptoms such as pain and pruritus.^55^ This is important because a greater severity of psoriatic symptoms leads to an increase in the levels of depressive^8^ and anxiety^9^ symptoms.

However, regardless of the physical factors, each person with psoriasis is likely to react differently to their illness‐related symptoms. According to the Common‐Sense Model of Self‐Regulation,^56^ beliefs patients hold about their illness (illness perceptions) play an important role in mental and physical health outcomes.^69^ To illustrate this, a patient with normal body weight who *perceives* their psoriasis as being more severe than a patient who is overweight is more likely to suffer from low self‐esteem, body image issues, and social isolation.^57^ Patients who *self*‐*assess* their psoriasis as severe are especially vulnerable to mental health issues.^58^, ^59^ Together these perceptions can stifle physical activity and motivation to engage in healthy behaviours,^60^ contributing to obesity. In a study, beliefs about psoriasis causing weight gain were linked to a higher BMI.^5^ Exploration of illness perceptions may reveal important information about how patients conceptualise their psoriasis and the consequences of this on their mental health and psoriasis‐related behaviours, as well as weight‐related behaviours.^61^, ^62^ Future studies should investigate these underlying cognitive and behavioural factors that may better explain the high rates of obesity in psoriasis, beyond the role of depression and anxiety.^7^

### 6.2 Strengths and limitations

To the best of our knowledge, this is the first study to look at the association between mental health and body weight, measured cross‐sectionally and 12 months later. Compared to previous studies,^7^ the main strengths of the study are its large sample size, the adjustment for several important demographic and illness‐related covariates, and the use of waist circumference as an outcome as a more accurate indication of body fat.

Despite the study's strengths, important limitations related to the use of real‐world data need to be considered.^63^ The analyses were restricted to the use of dichotomised variables of depression and anxiety, indicative of probable diagnostic levels of depression and/or anxiety. This approach is likely to have obscured the association between mental health with weight‐related outcomes due to reduced power.^66^ There was substantial amount of missing weight‐related outcome data (a typical characteristic of routine data) which reduced the analytical samples of patients.

Particularly, given the focus on mental health and weight here, as discussed earlier, non‐response and attrition biases are likely present. Although treatment type was included as a control variable within the analysis, we were unable to consider the evidence that some biologic treatments such as tumour necrosis factor alpha inhibitors, but not interleukin (IL)‐12/23, may be associated with increased body weight or BMI.^70^ Therefore, any possibly conflicting effects of different types of biologics on waist circumference or BMI remain future research's priority. In addition, the highly selective population sample drawn from a tertiary clinic with integrated psychological support may limit the findings' generalisability.

## 7 CONCLUSION

The lack of evidence linking mental health and body weight in this study suggests that obesity in psoriasis may be more directly related to comorbid conditions and severity of psoriasis. However, mental health in this study was limited to measures of probably anxiety and depression caseness. It is likely that more complex cognitive, behavioural, and emotional factors, including beliefs and perceptions about body weight, are relevant to understanding obesity in psoriasis. People living with obesity who deal with multimorbidity and greater disease severity may be more prone to psoriasis‐related unhelpful coping behaviours such as social withdrawal and emotional eating or be restricted in their physical activity due to disability. The coping and adjustment demands imposed by multimorbidity and greater disease severity, if unmet, may drive mental health issues and contribute to an increase in body weight. Future studies are needed to (i) confirm the findings using rigorously designed longitudinal studies, (ii) further investigate the impact of depression and anxiety on body weight with a focus to reduce attrition among people who live with mental health problems, and (iii) explore the role of beliefs and behaviours in the association between obesity and mental health in psoriasis.

## CONFLICT OF INTEREST

Catherine Smith has received departmental research funding from industries that manufacture treatments for psoriasis including AbbVie, Boehringer Ingelheim, Glaxo SmithKline, Leo, Pfizer, Novartis, Regeneron, and is an investigator within consortia that have industry partners (see biomap.eu and psort.org.uk).

## ETHICS STATEMENT

The research data used in this study has been granted ethical approval by the National Health Service (NHS) Research Ethics Committee in the United Kingdom (REC reference: 12/SC/0422) until 2023. Since IMPARTS data collection is part of routine clinical care, formal consent to participate is not required.

## AUTHOR CONTRIBUTIONS

**Neli T. Pavlova:** Conceptualization (lead); Data curation (lead); Formal analysis (lead); Investigation (lead); Methodology (lead); Project administration (equal); Resources (lead); Validation (lead); Writing ‐ original draft (lead); Writing – review & editing (lead). **Rona Moss‐Morris:** Conceptualization (equal); Data curation (equal); Formal analysis (equal); Investigation (equal); Methodology (equal); Resources (equal); Supervision (equal); Validation (equal); Writing – original draft (equal); Writing – review & editing (equal). **Catherine Smith:** Conceptualization (equal); Formal analysis (equal); Methodology (equal); Supervision (equal); Validation (equal); Writing – original draft (equal); Writing – review & editing (equal). **Ewan Carr:** Methodology (equal); Supervision (equal); Validation (equal); Writing – original draft (equal); Writing – review & editing (equal). **Lauren Rayner:** Conceptualization (equal); Data curation (equal); Formal analysis (equal); Methodology (equal); Supervision (equal); Writing – original draft (equal); Writing – review & editing (equal). **Federica Picariello:** Conceptualization (lead); Data curation (lead); Formal analysis (lead); Investigation (equal); Methodology (lead); Project administration (equal); Resources (equal); Supervision (lead); Validation (lead); Writing – original draft (lead); Writing – review & editing (lead).

## Supporting information

Supporting Information S1

## Data Availability

The data that support the findings are not publicly available due to containing information that could compromise the privacy of research participants.

## References

[1] Parisi R , Symmons DP , Griffiths CE , Ashcroft DM . Global epidemiology of psoriasis: a systematic review of incidence and prevalence. J Invest Dermatol. 2013;133(2):377–85. 23014338 10.1038/jid.2012.339

[2] Mahil S , McSweeney S , Kloczko E , McGowan B , Barker J , Smith C . Does weight loss reduce the severity and incidence of psoriasis or psoriatic arthritis? A critically appraised topic. Br J Dermatol. 2019;181(5):946–53. 30729517 10.1111/bjd.17741

[3] Iskandar I , Ashcroft D , Warren R , Yiu Z , McElhone K , Lunt M , et al. Demographics and disease characteristics of patients with psoriasis enrolled in the British Association of Dermatologists Biologic Interventions Register. Br J Dermatol. 2015;173 (2):510–8. 25989336 10.1111/bjd.13908

[4] Innamorati M , Quinto RM , Imperatori C , Lora V , Graceffa D , Fabbricatore M , et al. Health‐related quality of life and its association with alexithymia and difficulties in emotion regulation in patients with psoriasis. Compr Psychiatr. 2016;70:200–8. 10.1016/j.comppsych.2016.08.001 27565774

[5] Kim G , Seidler E , Kimball A . The relative impact of psoriasis and obesity on socioeconomic and medical outcomes in psoriasis patients. J Eur Acad Dermatol Venereol. 2014;28(2):216–21. 23347229 10.1111/jdv.12089

[6] Luppino FS , de Wit LM , Bouvy PF , Stijnen T , Cuijpers P , Penninx BW , et al. Overweight, obesity, and depression: a systematic review and meta‐analysis of longitudinal studies. Arch Gen Psychiatr. 2010;67(3):220–9. 20194822 10.1001/archgenpsychiatry.2010.2

[7] Pavlova N , Kioskli K , Smith C , Picariello F , Rayner L , Moss‐Morris R Psychosocial aspects of obesity in adults with psoriasis: a systematic review. Skin Health Dis. 2021. 10.1002/ski2.33 PMC9060108 35664982

[8] Payne M , Starr KP , Orenduff M , Mulder H , McDonald S , Spira A , et al. Quality of life and mental health in older adults with obesity and frailty: associations with a weight loss intervention. J Nutr Health Aging. 2018;22(10):1259–65. 30498835 10.1007/s12603-018-1127-0 PMC6444357

[9] Kouris A , Christodoulou C , Stefanaki C , Livaditis M , Tsatovidou R , Kouskoukis C , et al. Quality of life and psychosocial aspects in Greek patients with psoriasis: a cross‐sectional study. Anais Brasileiros Dermatol. 2015;90(6):841–5. 10.1590/abd1806-4841.20154147 PMC4689072 26734865

[10] Griffiths CEM , Jo SJ , Naldi L , Romiti R , Guevara‐Sangines E , Howe T , et al. A multidimensional assessment of the burden of psoriasis: results from a multinational dermatologist and patient survey. Br J Dermatol. 2018;179(1):173–81. 29328510 10.1111/bjd.16332

[11] Martínez‐Ortega JM , Nogueras P , Muñoz‐Negro JE , Gutiérrez‐Rojas L , González‐Domenech P , Gurpegui M . Quality of life, anxiety and depressive symptoms in patients with psoriasis: a case‐control study. J Psychosom Res. 2019;124:109780. 31443809 10.1016/j.jpsychores.2019.109780

[12] National Health Service (NHS) . What is the body mass index (BMI)? 2019.

[13] Luz RH , Barbosa AR , d'Orsi E . Waist circumference, body mass index and waist‐height ratio: are two indices better than one for identifying hypertension risk in older adults? Prev Med. 2016;93:76–81. 27663432 10.1016/j.ypmed.2016.09.024

[14] NICE The National Institute for Health and Care Excellence . Psoriasis: assessment and management. 2014. 32049470

[15] Speed MS , Jefsen OH , Børglum AD , Speed D , Østergaard SD . Investigating the association between body fat and depression via Mendelian randomization. Transl Psychiatry. 2019;9(1):1–9. 31383844 10.1038/s41398-019-0516-4 PMC6683191

[16] Takeshita J , Grewal S , Langan SM , Mehta NN , Ogdie A , van Voorhees AS , et al. Psoriasis and comorbid diseases: epidemiology. J Am Acad Dermatol. 2017;76(3):377–90. 28212759 10.1016/j.jaad.2016.07.064 PMC5731650

[17] Da Costa LA , Arora P , García‐Bailo B , Karmali M , El‐Sohemy A , Badawi A . The association between obesity, cardiometabolic disease biomarkers, and innate immunity‐related inflammation in Canadian adults. Diab Metab Syndrome Obes Targets Ther. 2012;5:347. 10.2147/DMSO.S35115 PMC3468056 23055759

[18] Golpour M , Hosseini SH , Khademloo M , Ghasemi M , Ebadi A , Koohkan F , et al. Depression and anxiety disorders among patients with psoriasis: a hospital‐based case‐control study. Dermatol Res Pract. 2012:2012. 10.1155/2012/381905 PMC3403219 22844272

[19] Gray CM , Anderson AS , Clarke AM , Dalziel A , Hunt K , Leishman J , et al. Addressing male obesity: an evaluation of a group‐based weight management intervention for Scottish men. J Mens Health. 2009;6(1):70–81.

[20] Herva A , Laitinen J , Miettunen J , Veijola J , Karvonen J , Läksy K , et al. Obesity and depression: results from the Longitudinal Northern Finland 1966 Birth Cohort Study. Int J Obes. 2006;30(3):520–7. 10.1038/sj.ijo.0803174 16302014

[21] Helmick CG , Lee‐Han H , Hirsch SC , Baird TL , Bartlett CL . Prevalence of psoriasis among adults in the US: 2003–2006 and 2009–2010 National Health and Nutrition Examination Surveys. Am J Prev Med. 2014;47(1):37–45. 24746373 10.1016/j.amepre.2014.02.012 PMC4653077

[22] Liao W , Luo Z , Hou Y , Cui N , Liu X , Huo W , et al. Age and gender specific association between obesity and depressive symptoms: a large‐scale cross‐sectional study. BMC Publ Health. 2020;20(1):1–10. 10.1186/s12889-020-09664-8 PMC7568408 33069213

[23] Morgan PJ , Callister R , Collins CE , Plotnikoff RC , Young MD , Berry N , et al. The SHED‐IT Community Trial: a randomized controlled trial of internet‐and paper‐based weight loss programs tailored for overweight and obese men. Ann Behav Med. 2013;45(2):139–52. 23129021 10.1007/s12160-012-9424-z

[24] Mazzeschi C , Pazzagli C , Buratta L , Reboldi GP , Battistini D , Piana N , et al. Mutual interactions between depression/quality of life and adherence to a multidisciplinary lifestyle intervention in obesity. J Clin Endocrinol Metab. 2012;97(12):E2261–E5. 23007686 10.1210/jc.2012-2364

[25] Somerset S , Graham L , Markwell K . Depression scores predict adherence in a dietary weight loss intervention trial. Clin Nutr. 2011;30(5):593–8. 21575998 10.1016/j.clnu.2011.04.004

[26] Rayner L , Matcham F , Hutton J , Stringer C , Dobson J , Steer S , et al. Embedding integrated mental health assessment and management in general hospital settings: feasibility, acceptability and the prevalence of common mental disorder. Gen Hosp Psychiatr. 2014;36(3):318–24. 10.1016/j.genhosppsych.2013.12.004 24630892

[27] Von Elm E , Altman DG , Egger M , Pocock SJ , Gøtzsche PC , Vandenbroucke JP . The Strengthening the Reporting of Observational Studies in Epidemiology (STROBE) statement: guidelines for reporting observational studies. Bull World Health Organ. 2007;85:867–72. 18038077 10.2471/BLT.07.045120 PMC2636253

[28] National Health Service (NHS) . Statistics on obesity, physical activity and diet. England; 2020. p. 2021.

[29] Kroenke K , Spitzer RL , Williams JB . The Patient Health Questionnaire‐2: validity of a two‐item depression screener. Med Care. 2003:1284–92. 14583691 10.1097/01.MLR.0000093487.78664.3C

[30] Kroenke K , Spitzer RL , Williams JB . The PHQ‐9: validity of a brief depression severity measure. J Gen Intern Med. 2001;16(9):606–13. 11556941 10.1046/j.1525-1497.2001.016009606.x PMC1495268

[31] Spitzer RL , Kroenke K , Williams JB , Löwe B . A brief measure for assessing generalized anxiety disorder: the GAD‐7. Arch Intern Med. 2006;166(10):1092–7. 16717171 10.1001/archinte.166.10.1092

[32] Warren RB , Halliday A , Graham CN , Gilloteau I , Miles L , McBride D . Secukinumab significantly reduces psoriasis‐related work impairment and indirect costs compared with ustekinumab and etanercept in the United Kingdom. J Eur Acad Dermatol Venereol. 2018;32(12):2178–84. 29846965 10.1111/jdv.15094 PMC6586050

[33] Gisondi P , Del Giglio M , Girolomoni G . Treatment approaches to moderate to severe psoriasis. Int J Mol Sci. 2017;18(11):2427. 29144382 10.3390/ijms18112427 PMC5713395

[34] Jensen P , Skov L . Psoriasis and obesity. Dermatology. 2016;232(6):633–9. 28226326 10.1159/000455840

[35] Manzel A , Muller DN , Hafler DA , Erdman SE , Linker RA , Kleinewietfeld M . Role of “Western diet” in inflammatory autoimmune diseases. Curr Allergy Asthma Rep. 2014;14(1):1–8. 10.1007/s11882-013-0404-6 PMC4034518 24338487

[36] Cohen AD , Vender R , Naldi L , Kalb RE , Torres T , Rajagopalan M , et al. Biosimilars for the treatment of patients with psoriasis: a consensus statement from the Biosimilar Working Group of the International Psoriasis Council. JAAD Int. 2020;1(2):224–30. 34409344 10.1016/j.jdin.2020.09.006 PMC8361899

[37] The International Psoriasis Council . Psoriasis severity reclassification project; 2021.

[38] Feldman S , Krueger G . Psoriasis assessment tools in clinical trials. Ann Rheumat Dis. 2005;64 (Suppl 2):ii65–ii8. 15708941 10.1136/ard.2004.031237 PMC1766877

[39] Donnelly JE , Blair SN , Jakicic JM , Manore MM , Rankin JW , Smith BK . American College of Sports Medicine Position Stand. Appropriate physical activity intervention strategies for weight loss and prevention of weight regain for adults. Med Sci Sports Exerc. 2009;41(2):459–71. 19127177 10.1249/MSS.0b013e3181949333

[40] StataCorp LP . Stata statistical software: release 16. College Station, TX: StataCorp L; 2015.

[41] Ross R , Neeland IJ , Yamashita S , Shai I , Seidell J , Magni P , et al. Waist circumference as a vital sign in clinical practice: a consensus statement from the IAS and ICCR Working Group on Visceral Obesity. Nat Rev Endocrinol. 2020;16(3):177–89. 32020062 10.1038/s41574-019-0310-7 PMC7027970

[42] Bronckers IMGJ , van Geel MJ , van deKerkhof PCM , de Jong EMGJ , Seyger MMB . A cross‐sectional study in young adults with psoriasis: potential determining factors in quality of life, life course and work productivity. J Dermatol Treat. 2018:1–8. 10.1080/09546634.2018.1506077 30102075

[43] Cohen BE , Martires KJ , Ho RS . Psoriasis and the risk of depression in the US population: National Health and Nutrition Examination Survey 2009‐2012. JAMA Dermatol. 2016;152(1):73–9. 26421371 10.1001/jamadermatol.2015.3605

[44] Inelmen EM , Sergi G , Coin A , Miotto F , Peruzza S , Enzi G . Can obesity be a risk factor in elderly people? Obes Rev. 2003;4(3):147–55. 12916816 10.1046/j.1467-789x.2003.00107.x

[45] Molina‐Molina E , Garruti G , Shanmugam H , Di Palo DM , Grattagliano I , Mastronuzzi T , et al. Aging and nutrition. Paving the way to better health. Roman J Int Med. 2020;58(2):55–68. 10.2478/rjim-2020-0005 32134741

[46] Walls HL , Stevenson CE , Mannan HR , Abdullah A , Reid CM , McNeil JJ , et al. Comparing trends in BMI and waist circumference. Obesity. 2011;19(1):216–9. 20559295 10.1038/oby.2010.149

[47] Stevens J , Katz EG , Huxley RR . Associations between gender, age and waist circumference. Eur J Clin Nutr. 2010;64(1):6–15. 19738633 10.1038/ejcn.2009.101 PMC5909719

[48] Agborsangaya CB , Ngwakongnwi E , Lahtinen M , Cooke T , Johnson JA . Multimorbidity prevalence in the general population: the role of obesity in chronic disease clustering. BMC Publ Health. 2013;13(1):1–6. 10.1186/1471-2458-13-1161 PMC4029057 24325303

[49] Tohid H , Aleem D , Jackson C . Major depression and psoriasis: a psychodermatological phenomenon. Skin Pharmacol Physiol. 2016;29(4):220–30. 27536769 10.1159/000448122

[50] Konttinen H , Männistö S , Sarlio‐Lähteenkorva S , Silventoinen K , Haukkala A . Emotional eating, depressive symptoms and self‐reported food consumption. A population‐based study. Appetite. 2010;54(3):473–9. 20138944 10.1016/j.appet.2010.01.014

[51] Konttinen H , Van Strien T , Männistö S , Jousilahti P , Haukkala A . Depression, emotional eating and long‐term weight changes: a population‐based prospective study. Int J Behav Nutr Phys Activ. 2019;16(1):1–11. 10.1186/s12966-019-0791-8 PMC6427874 30894189

[52] VanStrien T , Herman CP , Anschutz DJ , Engels RC , deWeerth C . Moderation of distress‐induced eating by emotional eating scores. Appetite. 2012;58(1):277–84. 22037008 10.1016/j.appet.2011.10.005

[53] Suls J , Green PA , Boyd CM . Multimorbidity: implications and directions for health psychology and behavioral medicine. Health Psychol. 2019;38(9):772. 31436463 10.1037/hea0000762 PMC6750244

[54] Zheng Q , Sun XY , Miao X , Xu R , Ma T , Zhang YN , et al. Association between physical activity and risk of prevalent psoriasis: a MOOSE‐compliant meta‐analysis. Medicine. 2018;97(27). 10.1097/MD.0000000000011394 PMC6076093 29979432

[55] Bahali AG , Onsun N , Su O , Ozkaya DB , Dizman D , Topukcu B , et al. The relationship between pruritus and clinical variables in patients with psoriasis. Anais Brasil Dermatol. 2017;92:470–3. 10.1590/abd1806-4841.20175402 PMC5595591 28954093

[56] Leventhal H , Meyer D , Nerenz D . The common sense representation of illness danger. Contrib Med Psychol. 1980;2:7–30.

[57] Price A , Goodwin L , Rayner L , Shaw E , Hansford P , Sykes N , et al. Illness perceptions, adjustment to illness, and depression in a palliative care population. J Pain Symptom Manag. 2012;43(5):819–32. 10.1016/j.jpainsymman.2011.05.013 22285286

[58] Lim DS , Bewley A , Oon HH . Psychological profile of patients with psoriasis. Ann Acad Med Singapore. 2018;47(12):516–22. 30636268

[59] Carr E , Mahil SK , Brailean A , Dasandi T , Pink AE , Barker JN , et al. Association of patient mental health status with the level of agreement between patient and physician ratings of psoriasis severity. JAMA Dermatol. 2021. 10.1001/jamadermatol.2020.5844 PMC7931137 33656512

[60] Pariser D , Schenkel B , Carter C , Farahi K , Brown TM , Ellis CN , et al. A multicenter, non‐interventional study to evaluate patient‐reported experiences of living with psoriasis. J Dermatol Treat. 2016;27(1):19–26. 10.3109/09546634.2015.1044492 PMC4732424 26138406

[61] Horne R , Weinman J . Self‐regulation and self‐management in asthma: exploring the role of illness perceptions and treatment beliefs in explaining non‐adherence to preventer medication. Psychol Health. 2002;17(1):17–32.

[62] French DP , Cooper A , Weinman J . Illness perceptions predict attendance at cardiac rehabilitation following acute myocardial infarction: a systematic review with meta‐analysis. J Psychosom Res. 2006;61(6):757–67. 17141663 10.1016/j.jpsychores.2006.07.029

[63] Hemkens LG , Contopoulos‐Ioannidis DG , Ioannidis JP . Routinely collected data and comparative effectiveness evidence: promises and limitations. Can Med Assoc J. 2016;188(8):E158–E64. 26883316 10.1503/cmaj.150653 PMC4868623

[64] Dupuis M , Strippoli MPF , Gholam‐Rezaee M , Preisig M , Vandeleur CL . Mental disorders, attrition at follow‐up, and questionnaire non‐completion in epidemiologic research. Illustrations from the CoLaus| PsyCoLaus study. Int J Methods Psychiatr Res. 2019;28(4):e1805. 31568629 10.1002/mpr.1805 PMC7027429

[65] Moroshko I , Brennan L , O'Brien P . Predictors of dropout in weight loss interventions: a systematic review of the literature. Obes Rev. 2011;12(11):912–34. 21815990 10.1111/j.1467-789X.2011.00915.x

[66] Fedorov V , Mannino F , Zhang R . Consequences of dichotomization. Pharmaceut Stat. 2009;8(1):50–61. 10.1002/pst.331 18389492

[67] Hanel G , Henningsen P , Herzog W , Sauer N , Schaefert R , Szecsenyi J , et al. Depression, anxiety, and somatoform disorders: vague or distinct categories in primary care? Results from a large cross‐sectional study. J Psychosom Res. 2009;67(3):189‐97. 19686874 10.1016/j.jpsychores.2009.04.013

[68] Kroenke K , Spitzer RL , Williams JB , Monahan PO , Löwe B . Anxiety disorders in primary care: prevalence, impairment, comorbidity, and detection. Ann Intern Med. 2007;146(5):317‐25. 17339617 10.7326/0003-4819-146-5-200703060-00004

[69] Hagger MS , Koch S , Chatzisarantis NL , Orbell S . The common sense model of self‐regulation: meta‐analysis and test of a process model. Psychol Bull. 2017;143(11):1117. 28805401 10.1037/bul0000118

[70] Wu M‐Y , Yu C‐L , Yang S‐J , Chi C‐C . Change in body weight and body mass index in psoriasis patients receiving biologics: a systematic review and network meta‐analysis. J Am Acad Dermatol. 2020;82(1):101–9. 31400455 10.1016/j.jaad.2019.07.103

