# Supplementary material for: The importance of illness severity and multimorbidity in the association between mental health and body weight in psoriasis: Cross‐sectional and longitudinal analysis
Source: Skin Health Dis. 2022 Apr 19;2(4):e117. doi: 10.1002/ski2.117 (PMC9720224; doi:10.1002/ski2.117)
Supplement: Supplementary file 1 — Supporting Information S1 [file SKI2-2-e117-s001.docx]

Supplementary Material A

Additional analysis

| Supplementary Table A1: Comparison of demographic and illness -related variables between patients with available waist circumference and those with missing waist circumference at time one and at 12 months follow up | | | | | | | | | | | |
| --- | --- | --- | --- | --- | --- | --- | --- | --- | --- | --- | --- |
|  | | **Time one**  **(the first available data entry)** | | | | | **Follow-up**  **(12 months later)** | | | | |
| Variable |  | **Waist circumference *present*** | **N** | **Wait circumference**  ***missing*** | **N** | **p-value** | **Waist circumference *present*** | **N** | **Wait circumference *missing*** | **N** | **p-value** |
| Gender, n (%) | Female | 49.8% | 128 | 50.2% | 129 | 0.008* | 59.0% | 69 | 41.0% | 48 | 0.367 |
|  | Male | 60.0% | 282 | 40.0% | 188 |  | 63.9% | 154 | 36.1% | 87 |  |
| Age (year), mean (SD) | | 46.04 (13.57) | 410 | 46.01 (12.83) | 317 | 0.0978 | 45.56 (12.65) | 223 | 45.33 (13.13) | 135 | 0.869 |
| Ethnicity n (%) | White | 57.1% | 333 | 42.9% | 250 | 0.383 | 62.5% | 178 | 37.5% | 107 | 0.898 |
|  | Black and Ethnic minorities | 53.1% | 77 | 46.9% | 68 |  | 61.6% | 45 | 38.4% | 28 |  |
| Treatment n (%) | Taking either biologic or systemic treatment, yes | 55.2% | 232 | 44.8% | 188 | 0.843 | 66.4% | 144 | 33.6% | 73 | 0.041* |
|  | No systemic treatment, yes | 56.0% | 154 | 44.0% | 121 |  | 55.4% | 72 | 44.6% | 58 |  |
| PASI, mean (SD) | | 4.67 (5.08) | 395 | 5.30 (5.25) | 308 | 0.106 | 3.44 (4.36) | 221 | 4.35 (4.50) | 135 | 0.06 |
| Total number of  comorbid medical conditions,  mean (SD) | | 0.93 (1.34) | 198 | 1.22 (1.21) | 115 | 0.058 | 0.93 (1.34) | 198 | 1.22 (1.21) | 115 | 0.058 |
| Depression (PHQ), n (%) | **Depressed** | 58.9% | 86 | 41.1% | 60 | 0.481 | 64.1% | 34 | 35.9% | 19 | 0.762 |
|  | **Not depressed** | 55.7% | 324 | 44.3% | 277 |  | 62.0% | 189 | 38.0% | 116 |  |
| Anxiety (GAD), n (%) | **Anxious** | 56.3% | 71 | 43.7% | 49 | 0.491 | 75.0% | 30 | 25.0% | 10 | 0.078 |
|  | Not anxious | 55.8% | 339 | 44.2% | 269 |  | 60.7% | 193 | 39.3% | 125 |  |

| Supplementary Table A2: Comparison of demographic and illness -related variables between patients with available BMI and those with missing BMI at time one and at 12 months follow up | | | | | | | | | | | |
| --- | --- | --- | --- | --- | --- | --- | --- | --- | --- | --- | --- |
|  | | **Time one**  **(the first available data entry)** | | | | | **Follow-up**  **(12 months later)** | | | | |
| Variable |  | BMI *present* | N | BMI *missing* | N | p-value | BMI *present* | N | BMI *missing* | N | p-value |
| Gender, n (%) | Female | 65.4% | 168 | 34.6% | 89 | 0.873 | 76.1% | 89 | 23.9% | 28 | 0.624 |
|  | Male | 66.0% | 310 | 34.0% | 160 |  | 76.8% | 185 | 23.2% | 56 |  |
| Age (year),  mean (SD) | | 45.66 (13.29) | 478 | 46.73 (13.15) | 249 | 0.301 | 45.33 (12.98) | 274 | 45.92(12.39) | 84 | 0.715 |
| Ethnicity,  n (%) | White | 34.0% | 198 | 66.0% | 385 | 0.666 | 75.8% | 216 | 24.2% | 69 | 0.510 |
|  | Black and Ethnic minorities | 35.9% | 52 | 64.1% | 93 |  | 79.5% | 58 | 20.5% | 15 |  |
| Treatment,  n (%) | Taking either biologic or systemic treatment, yes | 64.3% | 270 | 35.7% | 150 | 0.19 | 72.8% | 158 | 27.2% | 59 | 0.03* |
|  | No systemic treatment, yes | 69.1% | 190 | 30.9% | 85 |  | 83.1% | 108 | 16.9% | 22 |  |
| PASI, mean (SD) | | 5.30 (5.81) | 468 | 4.76 (4.80) | 235 | 0.189 | 3.67 (4.01) | 272 | 4.15 (5.58) | 84 | 0.385 |
| Total number  of comorbid medical  conditions, mean (SD) | | 1.02 (1.32) | 421 | 0.83 (1.22) | 224 | 0.08 | 1.10 (1.35) | 240 | 0.84 (1.1) | 73 | 0.134 |
| Depression (PHQ), n (%) | Depressed | 44.5% | 65 | 55.5% | 81 | 0.004* | 79.3% | 42 | 20.7% | 11 | 0.614 |
|  | Not depressed | 68.2% | 397 | 31.8% | 185 |  | 76.1% | 232 | 23.9% | 73 |  |
| Anxiety (GAD), n (%) | Anxious | 60.0% | 72 | 40.0% | 48 | 0.153 | 80.0% | 32 | 20.0% | 8 | 0.583 |
|  | Not anxious | 66.8% | 406 | 33.2% | 202 |  | 76.1% | 242 | 23.9% | 76 |  |

| Supplementary Table A3: Exploratory bivariate associations between predictors and outcomes at time one and at 12 months follow-up | | | | |
| --- | --- | --- | --- | --- |
| Predictors | **Outcomes at time one** | | **Outcomes at 12 months follow up** | |
|  | **BMI** | **Waist circumference** | **BMI** | **Waist circumference** |
| Age | r= 0.20, p<0.001* | r= 0.20, p<0.001* | r= 0.20, p= 0.0009* | r= 0.34, p< 0.001* |
| Gender | r= 0.02, p= 0.679 | r= 0.18, p= 0.0002* | r= 0.06, p= 0.316 | r= 0.25, p= 0.0002* |
| Ethnicity | r= 0.07, p= 0.147 | r= 0.03, p= 0.489 | r= 0.09, p= 0.12 | r= 0.12, p= 0.06 |
| Treatment type | r= -0.06, p= 0.217 | r= -0.05, p= 0.361 | r= 0.04, p= 0.503 | r= -0.06, p= 0.355 |
| Number of comorbidities | r= 0.24, p<0.001* | r= 0.23, p<0.001* | r= .35, p< 0.001* | r= 0.22, p< 0.001* |
| PASI scores | r= 0.05, p= 0.244 | r= 0.06, p=0.247 | r= 0.19, p= 0.004* | r= 0.06, p= 0.35 |
| Depression | r= 0.01, p=0.908 | r= 0.09, p=0.06 | r= 0.08, p= 0.21 | r= 0.04, p= 0.52 |
| Anxiety | r= -0.03, p=0.583 | r= 0.074, p= 0.131 | r= 0.001, p= 0.979 | r= 0.10, p= 0.113 |

| **Supplementary Table A4: Depression and anxiety at time one and waist circumference at time one, using gender as moderator after controlling for demographic and illness-related variables** | | | | | |
| --- | --- | --- | --- | --- | --- |
| *Step and variable* | *B*  (standard error) | Significance level (2-tailed) | 95% Confidence Interval* | | R² Change |
|  |  |  | Lower | Upper |  |
| **N= 326** | | | | | |
| **Gender, male** | **8.22**  **(2.15)** | **≤0.001*** | **4.00** | **12.44** | 14.72% |
| Depression, present | -0.84 (6.75) | 0.902 | -14.12 | 12.45 |  |
| Gender, male  Depression, present | 3.52  (8.41) | 0.676 | -13.02 | 20.06 |  |
| Anxiety, present | -1.08  (7.24) | 0.881 | -15.33 | 13.17 |  |
| Gender, male  Anxiety, present | -7.20  (11.50) | 0.531 | -29.82 | 15.41 |  |
| **Age** | **0.20**  **(0.07)** | **0.006*** | **0.06** | **0.34** |  |
| Ethnicity, white present | 1.87  (2.16) | 0.387 | -2.38 | 6.13 |  |
| **Number of comorbidities** | **2.13**  **(0.72)** | **0.004*** | **0.70** | **3.55** |  |
| Psoriasis systemic treatment | 1.47  (1.78) | 0.411 | -2.04 | 4.97 |  |
| PASI | 0.14  (0.22) | 0.538 | -0.30 | 0.57 |  |

| **Supplementary Table A5: Depression and anxiety at time one and BMI at time one, using gender as moderator after controlling for demographic and illness-related variables** | | | | | |
| --- | --- | --- | --- | --- | --- |
| *Step and variable* | *B*  (standard error) | Significance level (2-tailed) | 95% Confidence Interval* | | R² Change |
|  |  |  | Lower | Upper |  |
| **N= 399** | | | | | |
| Gender, male | 0.32  (0.67) | 0.414 | -2.79 | 6.76 | 11.29% |
| Depression, present | 1.99  (2.43) | 0.635 | -1.00 | 1.63 |  |
| Gender, male  Depression, present | -1.30  (3.00) | 0.665 | -7.21 | 4.61 |  |
| Anxiety, present | -1.36  (2.56) | 0.594 | -6.39 | 3.66 |  |
| Gender, male  Anxiety, present | -0.73  (3.56) | 0.837 | -7.73 | 6.27 |  |
| Age | 0.06  (0.02) | 0.006* | 0.02 | 0.11 |  |
| Ethnicity, white present | 0.76  (0.70) | 0.275 | -0.61 | 2.13 |  |
| **Number of comorbidities** | **0.87**  **(0.23)** | **≤0.001*** | **0.41** | **1.32** |  |
| Psoriasis systemic treatment | 0.88  (0.58) | 0.130 | -0.26 | 2.02 |  |
| PASI | 0.09  (0.08) | 0.223 | -0.06 | 0.24 |  |

| **Supplementary Table A6: Depression and anxiety at the first data entry and waist circumference at 12 months follow up, using gender as moderator after controlling for demographic and illness-related variables** | | | | | |
| --- | --- | --- | --- | --- | --- |
| *Step and variable* | *B*  (standard error) | Significance level (2-tailed) | 95% Confidence Interval* | | R² Change |
|  |  |  | Lower | Upper |  |
| **N= 191** | | | | | |
| **Gender, male** | **6.74**  **(2.47)** | **0.007*** | **1.87** | **11.61** | 30.95% |
| Depression, present | 1.30  (8.07) | 0.872 | -14.63 | 17.24 |  |
| Gender, male  Depression, present | -3.16  (11.49) | 0.784 | -25.85 | 19.52 |  |
| Anxiety, present | 2.83  (8.04) | 0.725 | -13.04 | 18.70 |  |
| Gender, male  Anxiety, present | -14.53  (13.01) | 0.266 | -40.21 | 11.14 |  |
| Age | 0.27  (0.09) | 0.337 | -18.82 | 54.62 |  |
| Ethnicity, white present | 4.63  (2.54) | 0.070 | -0.38 | 9.63 |  |
| **Number of comorbidities** | **2.57**  **(0.86)** | **0.003*** | **0.88** | **4.27** |  |
| Psoriasis systemic treatment | 0.19  (2.19) | 0.931 | -4.14 | 4.52 |  |
| **PASI** | **1.19**  **(0.28)** | **≤0.001*** | **0.64** | **1.74** |  |

| **Supplementary Table A7: Depression and anxiety at the first data entry and BMI at 12 months follow up, using gender as moderator after controlling for demographic and illness-related variables** | | | | | |
| --- | --- | --- | --- | --- | --- |
| *Step and variable* | *B*  (standard error) | Significance level (2-tailed) | 95% Confidence Interval* | | R² Change |
|  |  |  | Lower | Upper |  |
| **N= 231** | | | | | |
| Gender, male | 0.62  (0.94) | 0.512 | -1.24 | 2.48 | 10.23% |
| Depression, present | 0.43  (2.48) | 0.862 | -4.45 | 5.32 |  |
| Gender, male  Depression, present | -4.33  (3.56) | 0.226 | -11.35 | 2.69 |  |
| Anxiety, present | -1.59  (3.42 | 0.641 | -8.33 | 5.14 |  |
| Gender, male  Anxiety, present | -2.27  (6.98) | 0.746 | -16.02 | 11.49 |  |
| Age | 0.05  (0.03) | 0.102 | -0.01 | 0.12 |  |
| Ethnicity, white present | 1.15  (0.94) | 0.222 | -0.70 | 2.30 |  |
| **Number of comorbidities** | **0.70**  **(0.33)** | **0.033*** | **0.06** | **1.35** |  |
| Psoriasis systemic treatment | 0.65  (0.81) | 0.420 | -0.94 | 2.24 |  |
| PASI | 0.12  (0.11) | 0.268 | -0.09 | 0.34 |  |

| Supplementary Table A8: Comparison on multimorbidity and PASI between patients with or without depression and anxiety at time one | | | | | | |
| --- | --- | --- | --- | --- | --- | --- |
|  | **Depressed**  ***(N=117)*** | **Not depressed**  ***(N= 528)*** | **p** | **Anxious**  ***(N=97)*** | **Not anxious**  ***(N=548)*** | ***p*** |
| Number of comorbidities  Mean, SD | M= 0.71,  SD= 1.23 | M= 1.00,  SD= 1.30 | 0.02* | M= 0.66,  SD= 1.23 | M= 1.00,  SD= 1.29 | 0.01* |
|  | **Depressed**  ***(N=139)*** | **Not depressed**  ***(N= 564)*** | **p** | **Anxious**  ***(N=116)*** | **Not anxious**  ***(N=587)*** | ***p*** |
| PASI  Mean, SD | M= 6.68,  SD= 0.58 | M= 4.52,  SD= 0.19 | p≤ 0.001* | M= 6.23,  SD= 0.59 | M= 4.69,  SD= 0.20 | 0.003* |

**Table Legend**

[Supplementary Table A1: Comparison of demographic and illness -related variables between patients with available waist circumference and those with missing waist circumference at time one and at 12 months follow up 2](#_Toc95453442)

[Supplementary Table A2: Comparison of demographic and illness -related variables between patients with available BMI and those with missing BMI at time one and at 12 months follow up 3](#_Toc95453443)

[Supplementary Table A3: Exploratory bivariate associations between predictors and outcomes at time one and at 12 months follow-up 4](#_Toc95453444)

[Supplementary Table A4: Depression and anxiety at time one and waist circumference at time one, using gender as moderator after controlling for demographic and illness-related variables 5](#_Toc95453445)

[Supplementary Table A5: Depression and anxiety at time one and BMI at time one, using gender as moderator after controlling for demographic and illness-related variables 6](#_Toc95453446)

[Supplementary Table A6: Depression and anxiety at the first data entry and waist circumference at 12 months follow up, using gender as moderator after controlling for demographic and illness-related variables 7](#_Toc95453447)

[Supplementary Table A7: Depression and anxiety at the first data entry and BMI at 12 months follow up, using gender as moderator after controlling for demographic and illness-related variables 8](#_Toc95453448)

[Supplementary Table A8: Comparison on multimorbidity and PASI between patients with or without depression and anxiety at time one 9](#_Toc95453449)

Supplementary Material B

eMethods

**Patient Health Questionnaire (PHQ) and Generalised Anxiety Disorder Scale (GAD)**

All participants completed PHQ‐21 and GAD‐22, consisting of the first two items of PHQ‐9 and GAD‐7, respectively. Participants answering positively (‘More than half the days’ or ‘Nearly every day’) to at least one item went on to complete the remaining items of the corresponding measure. Probable major depressive disorder (MDD; ‘depression’ in the manuscript) was defined in accordance with the DSM-V criteria3 as (i) responding positively to one of the first two items (‘Little interest or pleasure in doing things’, ‘Feeling down, depressed, or hopeless’); and (ii) responding positively to a minimum of five other depression symptoms. If one of the reported symptoms was suicidality (“Thoughts that you would be better off dead, or of hurting yourself”), an answer of ‘Several days or more’ often would indicate a positive response. Probable generalized anxiety disorder (GAD; ‘anxiety’ in the manuscript) was defined as (i) responding positively to one of the first two items (“Feeling nervous, anxious, or on edge”, or “Not being able to stop or control worrying”); and (ii) a total score of ≥10 (out of a total of 21).

**eReferences**

1. Kroenke K, Spitzer R, Williams J. The Patient Health Questionnaire-2: Validity of a Two-Item

Depression Screener. *Med Care*. 2003;41(11):10.

1. Spitzer RL, Kroenke K, Williams JBW, Löwe B. A Brief Measure for Assessing Generalized

Anxiety Disorder: The GAD-7. *Arch Intern Med*. 2006;166(10):1092-1097.

doi:10.1001/archinte.166.10.1092

1. American Psychiatric Association. *Diagnostic and Statistical Manual of Mental Disorders*. 5th

edition. American Psychiatric Association; 2013.

Supplementary Material C

Strengthening the Reporting of Observational Studies in Epidemiology

(STROBE) checklist

STROBE Statement—checklist of items that should be included in reports of observational studies

|  | Item No. | Recommendation | Page  No. | Relevant text from manuscript |
| --- | --- | --- | --- | --- |
| **Title and abstract** | 1 | (*a*) Indicate the study’s design with a commonly used term in the title or the abstract | 1 | The importance of illness severity and multimorbidity in the association between mental health and body weight in psoriasis: cross-sectional and longitudinal analysis |
|  |  | (*b*) Provide in the abstract an informative and balanced summary of what was done and what was found | 2-3 |  |
| Introduction | | | |  |
| Background/rationale | 2 | Explain the scientific background and rationale for the investigation being reported | 3-5 |  |
| Objectives | 3 | State specific objectives, including any prespecified hypotheses | 5 |  |
| Methods | | | |  |
| Study design | 4 | Present key elements of study design early in the paper | 6 |  |
| Setting | 5 | Describe the setting, locations, and relevant dates, including periods of recruitment, exposure, follow-up, and data collection | 6 |  |
| Participants | 6 | (*a*) *Cohort study*—Give the eligibility criteria, and the sources and methods of selection of participants. Describe methods of follow-up  *Cross-sectional study*—Give the eligibility criteria, and the sources and methods of selection of participants | 6 |  |
|  |  |  |  |  |
| Variables | 7 | Clearly define all outcomes, exposures, predictors, potential confounders, and effect modifiers. Give diagnostic criteria, if applicable | 7-8 |  |
| Data sources/ measurement | 8 | For each variable of interest, give sources of data and details of methods of assessment (measurement). Describe comparability of assessment methods if there is more than one group | 7-8 |  |
| Bias | 9 | Describe any efforts to address potential sources of bias | 8 | First, to investigate the impact of missing data, patients with and without outcome data (waist circumference and BMI) at time one and follow-up were compared for demographic, illness-related, and mental health variables |
| Study size | 10 | Explain how the study size was arrived at | 6 | To maximise the use of available data, three analytical samples were defined (Table 1) all of which had complete data on either BMI or waist circumference, on covariates (e.g., demographic, illness-related), and mental health variables (depression and anxiety). Patients with incomplete data on BMI or waist circumference, covariates (e.g., demographic, illness-related), or mental health variables (depression and anxiety) were excluded from the analytical samples. |

Continued on next page

| Quantitative variables | 11 | Explain how quantitative variables were handled in the analyses. If applicable, describe which groupings were chosen and why | 7 |  |
| --- | --- | --- | --- | --- |
| Statistical methods | 12 | (*a*) Describe all statistical methods, including those used to control for confounding | 8-9 |  |
|  |  | (*b*) Describe any methods used to examine subgroups and interactions | 8-9 |  |
|  |  | (*c*) Explain how missing data were addressed | 8-9 |  |
|  |  | (*d*) *Cohort study*—If applicable, explain how loss to follow-up was addressed  *Cross-sectional study*—If applicable, describe analytical methods taking account of sampling strategy |  |  |
| Results | | | | |
| Participants | 13* | (a) Report numbers of individuals at each stage of study—eg numbers potentially eligible, examined for eligibility, confirmed eligible, included in the study, completing follow-up, and analysed | 9 |  |
|  |  | (b) Give reasons for non-participation at each stage | N/A |  |
|  |  | (c) Consider use of a flow diagram | Table 2. |  |
| Descriptive data | 14* | (a) Give characteristics of study participants (eg demographic, clinical, social) and information on exposures and potential confounders | 9-10 |  |
|  |  | (b) Indicate number of participants with missing data for each variable of interest | 10 |  |
|  |  | (c) *Cohort study*—Summarise follow-up time (eg, average and total amount) | N/A |  |
| Outcome data | 15* | *Cohort study*—Report numbers of outcome events or summary measures over time | 12-13 |  |
|  |  | *Cross-sectional study—*Report numbers of outcome events or summary measures | 12-13 |  |
| Main results | 16 | (*a*) Give unadjusted estimates and, if applicable, confounder-adjusted estimates and their precision (eg, 95% confidence interval). Make clear which confounders were adjusted for and why they were included | 12-13 |  |
|  |  | (*b*) Report category boundaries when continuous variables were categorized | 12-13 |  |
|  |  | (*c*) If relevant, consider translating estimates of relative risk into absolute risk for a meaningful time period | N/A |  |

Continued on next page

| Other analyses | 17 | Report other analyses done—eg analyses of subgroups and interactions, and sensitivity analyses | 13 |  |
| --- | --- | --- | --- | --- |
| Discussion | | | | |
| Key results | 18 | Summarise key results with reference to study objectives | 13-14 |  |
| Limitations | 19 | Discuss limitations of the study, taking into account sources of potential bias or imprecision. Discuss both direction and magnitude of any potential bias | 16 |  |
| Interpretation | 20 | Give a cautious overall interpretation of results considering objectives, limitations, multiplicity of analyses, results from similar studies, and other relevant evidence | 14-15 |  |
| Generalisability | 21 | Discuss the generalisability (external validity) of the study results | 15-17 |  |
| Other information | |  | | |
| Funding | 22 | Give the source of funding and the role of the funders for the present study and, if applicable, for the original study on which the present article is based | 1 | This paper represents independent research part-funded by the National Institute for Health Research (NIHR) Maudsley Biomedical Research Centre at South London and Maudsley NHS Foundation Trust and King’s College London and NIHR Biomedical Research Centre (BRC) at Kings College London and Guys and St Thomas NHS Foundation Trust. The views expressed are those of the author(s) and not necessarily those of the NHS, the NIHR or the Department of Health and Social Care. |
